# Association Between Maternal Diabetes and ChildhoodNeurodevelopmental Disorders

**DOI:** 10.1192/j.eurpsy.2024.936

**Published:** 2024-08-27

**Authors:** K. Shah, C. Thomas

**Affiliations:** ^1^Wake Forest University, Winston-Salem; ^2^East Tennessee State University, Mountain Home, United States

## Abstract

**Introduction:**

The prevalence of metabolic disorders is rising, diabetes prevalence doubled during 1990-2017. In 2020, 7.8% of US pregnancies were complicated by Gestational Diabetes Mellitus (GDM). Our aim is to assess the impact this increase has on childhood developmental disorders and understand the link between maternal metabolic disorders and neurodevelopmental disorders in children with focus on Autism Spectrum Disorder (ASD) and ADHD.

**Objectives:**

Our aim is to assess the impact this increase has on childhood developmental disorders and understand the link between maternal metabolic disorders and neurodevelopmental disorders in children with focus on Autism Spectrum Disorder (ASD) and ADHD.

**Methods:**

A literature search was conducted using medical subject heading (MeSH) terms in PubMed, database from Jan 1 2014 to Feb 15, 2023. Only large-scale (n>20,000) were reviewed. A total of 3 articles were included in our final qualitative synthesis review.

**Results:**

An increased rate of ASD and ADHD are observed in children of mothers with insulin resistance, demonstrated by Type 2 Diabetes (T2DM) and GDM (Kuan-Ru Chen, et al.). T2DM had the strongest association with ASA and ADHD when looking at other neurodevelopmental disorders (Chen, et al.). GDM severity correlates to increased risk of ADHD (Xiang, et al.). Maternal obesity as a risk factor for ASA and ADHD has confidence intervals in the same ranges as immune dysregulatory disorders including Asthma and Autoimmune disorders (Woolfenden, et al.).

**Conclusions:**

Pathomechanism of neurodevelopmental disorders involves maternal oxidative stress and inflammation. Maternal T2DM and obesity are pro-inflammatory states that can be targeted as modifiable risk factors of ASD and ADHD in children. Preconception metabolic optimization and tight glycemic control in pregnancy are two ways clinicians can start to address the rates of rising ASD and ADHD.

**Disclosure of Interest:**

None Declared

